# Eastern Mediterranean Economic Exchange during the Iron Age: Portable X-Ray Fluorescence and Neutron Activation Analysis of Cypriot-Style Pottery in the Amuq Valley, Turkey

**DOI:** 10.1371/journal.pone.0166399

**Published:** 2016-11-30

**Authors:** Steven Karacic, James F. Osborne

**Affiliations:** 1 Department of Classics, Florida State University, Tallahassee, Florida, United States of America; 2 Department of Near Eastern Languages and Civilizations, Oriental Institute, University of Chicago, Chicago, Illinois, United States of America; New York State Museum, UNITED STATES

## Abstract

Two markers of regional exchange in the Eastern Mediterranean during the first millennium BCE are the White Painted and Bichrome Wares from Cyprus’s Cypro-Geometric and Cypro-Archaic periods. Although these ceramics are often assumed to be imports from Cyprus, excavations in southern Turkey at sites such as Tarsus-Gözlükule, Kilise Tepe, Sirkeli Höyük, and Kinet Höyük suggest that at least some of this pottery was produced locally, requiring a major revision of our understanding of economic interaction in the Eastern Mediterranean. We employ a combination of portable x-ray fluorescence and neutron activation analysis to investigate the White Painted and Bichrome Wares recovered from Tell Tayinat, Çatal Höyük, and Tell Judaidah, three sites in the Amuq Valley of southeastern Anatolia. Our results demonstrate that a clear geochemical distinction exists between imported and local versions of this pottery. Through comparison with legacy datasets, we locate the likely origin of the imported pottery in the Circum-Troodos sediments of central and southern Cyprus. The secondary and tertiary settlements of Çatal Höyük and Tell Judaidah had access only to this imported material. In contrast, the inhabitants of Tell Tayinat, capital city of the region, consumed both imported and locally produced White Painted and Bichrome Wares. This pattern cannot be explained in purely economic terms whereby the frequency of imports decreases as distance from the point of production increases. Instead, we suggest that elite feasting practices drove demand, resulting in either local potters producing Cypriot-style pottery or Cypriot potters settling in the vicinity of Tell Tayinat. These findings offer new insights into the relationship between historically attested Iron Age kingdoms in southern Turkey and Cyprus and complicate our understanding of exchange in the Eastern Mediterranean during the Iron Age.

## Introduction

As one of the most ubiquitous categories of ancient material culture, ceramics have long provided archaeologists with a tool to address a diverse suite of research questions, ranging from site chronology to local kinship patterns [1,2]. One well established use of ceramics is to determine patterns of ancient interaction, including economic interaction, by ascertaining the geographic movement of pottery from the place where it was produced to its final destination where it was excavated. Whether they were objects traded in their own right or, strictly speaking, for their contents, pottery vessels from locations more distant than the clay exploitation zone of local potters serve as powerful proxy indicators of exchange between regions.

Determining the place of origin for ceramics can be performed macroscopically by means of identifying stylistic or morphological outliers that resemble closely ceramic traditions from regions beyond one’s research area. An alternative, and often more data-driven, means of associating pottery with specific foreign locales is through geochemical provenience studies, which compare the elemental composition of an excavated pot with that of ceramic vessels or actual clay sources from other areas. Such studies rely on the assumption that different clay sources produce identifiably distinct geochemical signatures, with the variation between them greater than the variation within a single source [3]. A diverse range of analytical options exists for geochemical studies, typically increasing in power and resolution with the destructive nature of the method.

In this study, we perform provenience analysis on a group of putatively Cypriot ceramics of the early first millennium BCE, White Painted and Bichrome Wares from the Cypro-Geometric III to Cypro-Archaic I periods (ca. 850–600 BCE), that were excavated in the Amuq Valley of southeastern Anatolia. This project addresses three related research questions: (1) given a growing body of evidence that complicates previous assumptions about ceramic provenance in the Eastern Mediterranean, what is the proportion of White Painted and Bichrome Wares in the Amuq Valley that are genuinely imported from Cyprus; (2) what was the nature of economic exchange between the island of Cyprus and southeastern Anatolia during the Iron Age; and (3) what might be gleaned about the organization of craft production within local mainland Iron Age communities.

Because the objects are part of a study collection housed in the Oriental Institute Museum of the University of Chicago, we developed a methodology that began with non-destructive, but comparatively low resolution, portable x-ray fluorescence (pXRF) on all available ceramic sherds, in the process discovering two major geochemical groups. These results were confirmed in the second stage of our analysis, in which we sent representative samples of the identified geochemical groups to the Archaeometry Laboratory at the University of Missouri Research Reactor (MURR) for neutron activation analysis (NAA), a destructive but high resolution method. With our initial findings corroborated we then moved to a third stage of analysis in which we compared our results with previously analyzed ceramics and sediment samples from the Amuq Valley, Cilicia, and Cyprus. Our findings indicate that while White Painted and Bichrome vessels from secondary and tertiary sites in the Amuq Valley were likely produced on the island of Cyprus, the capital city both imported Cypriot pottery as well as produced a local version of it that is macroscopically indistinguishable from the imports. We suggest that demand from an elite class of administrators may account for this pattern. These findings index a complex economic and social relationship between a local southeastern Anatolian kingdom, Patina, and a contemporary polity, or polities, on Cyprus.

## Archaeological and Historical Background

**Fig 1:**
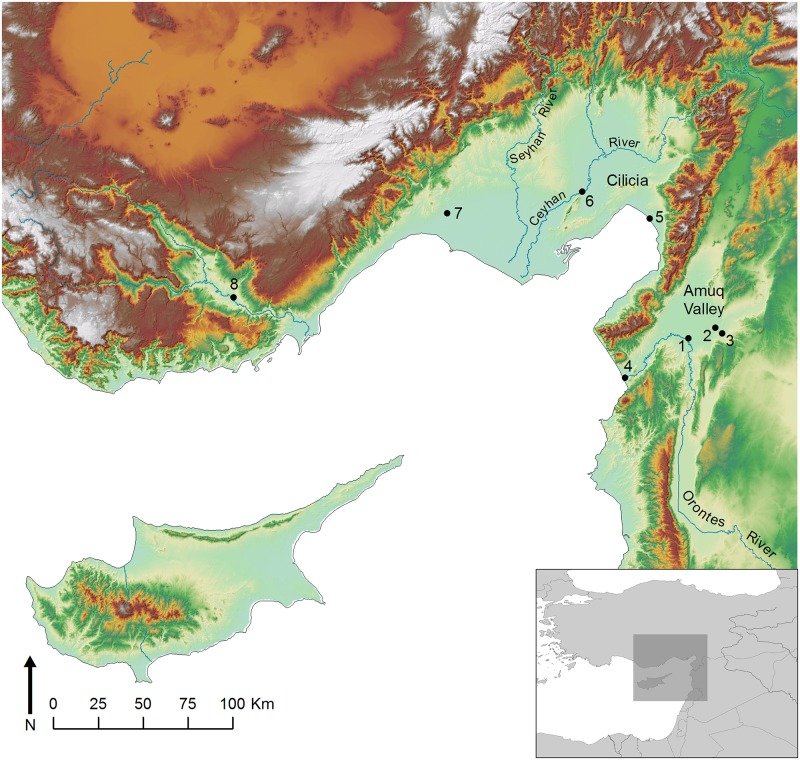
The northeast corner of the Mediterranean Sea and the island of Cyprus, with sites mentioned in the text. (1) Tell Tayinat; (2) Çatal Höyük; (3) Tell Judaidah; (4) Al Mina; (5) Kinet Höyük; (6) Sirkeli Höyük; (7) Tarsus-Gözlükale; (8) Kilise Tepe.

The Amuq Valley is a broad plain located roughly 40 km inland from the northeast corner of the Mediterranean Sea at the northern end of the Dead Sea Transform fault system (Fig 1). The alluvium deposited in the valley by the annually flooding Orontes River combined with annual precipitation averaging 600 mm has produced a landscape well suited for agricultural sedentary occupation [4], and as a result the Amuq is one of the densest archaeological regions in the Near East from the Neolithic period until the present [5,6]. Occupation during the Bronze and Iron Ages in particular, during which the Amuq witnessed the rise of the state and was home to a sequence of historically attested kingdoms, created a landscape of human-constructed mounds, or tells, that marked long-term places of settlement [7].

During the Iron Age II period, ca. 950–725 BCE, the Amuq Valley was ruled by the kingdom of Patina, one of several Syro-Anatolian city-states that existed from the Anatolian plateau to northeastern Syria [8–10]. All of these kingdoms were conquered, mostly during the mid-late eighth century BCE, by the Neo-Assyrian Empire based in northern Mesopotamia. Patina itself fell to the Assyrian ruler Tiglath-pileser III in 738 BCE [11]. At this time, fifty of the tells in the valley were occupied, plus another five in the delta of the Orontes River [12,13]. Site size modeling, nearest neighbor analysis, excavation data, and contemporary historical inscriptions combine to present a scenario in which Patina, and likely its neighboring Syro-Anatolian city-states as well, was characterized by a three-tiered settlement hierarchy: a high number of tertiary rural villages surrounded a small number of secondary centers, all of which were dominated by the capital city of Kunulua, the archaeological site of Tell Tayinat [12].

Sites of each of these three tiers were subjected to archaeological excavation in the 1930s by the Syrian-Hittite Expedition of the University of Chicago’s Oriental Institute; in addition to Tell Tayinat (35 ha), the expedition also explored Çatal Höyük (10 ha), a fortified secondary center, and Tell Judaidah (3 ha), an unfortified village. All three of these sites produced remains from multiple periods, including the early first millennium BCE, such that a complete portrait of the site hierarchy of the Iron Age kingdom of Patina was obtained [14–16]. Excavated material was shared between the local Hatay Archaeology Museum and the Oriental Institute Museum. Among the latter is a representative sample of the ceramic repertoires from each site, including Iron Age pottery imported to Patina from the island of Cyprus, roughly 200 km off the coast.

**Fig 2:**
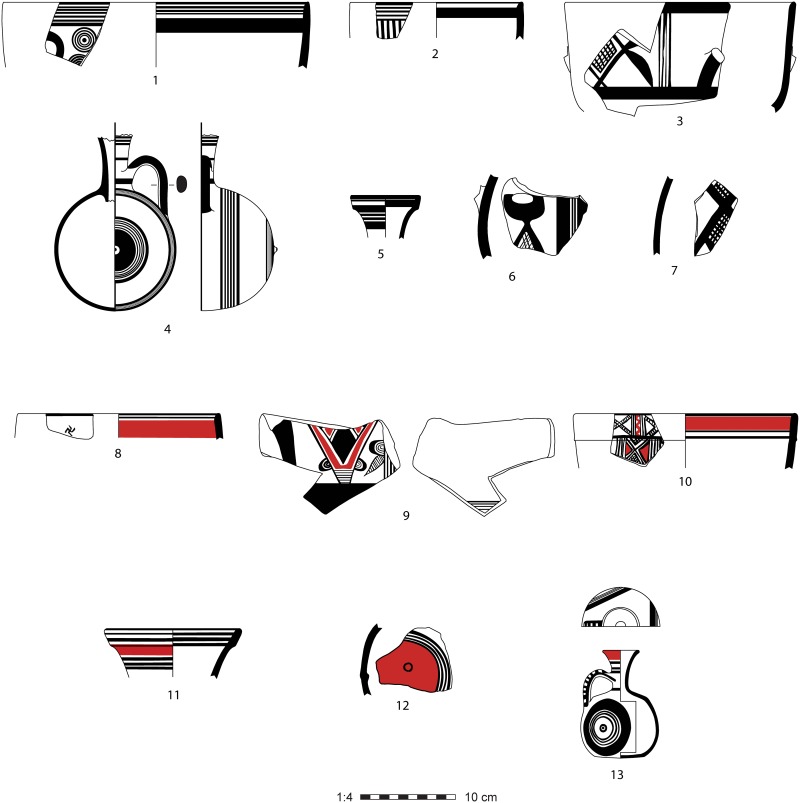
Cypro-Geometric III and Cypro-Archaic I (ca. 850–600 BCE) pottery from Tell Tayinat, ancient Kunulua. (1–3) White Painted Ware vertical-sided bowls; (4–7) White Painted Ware barrel jugs; (8–10) Bichrome Ware vertical-sided bowls; (11–12) Bichrome Ware barrel jugs; (13) Bichrome Ware juglet.

Contemporary with the mainland Syro-Anatolian city-states, a number of small kingdoms also existed on Cyprus during the early first millennium. The island’s Iron Age pottery sequence was first systematized by Gjerstad [17], whose dating scheme includes three sub-periods roughly coeval with the floruit of the Syro-Anatolian kingdoms and their subsequent Assyrian occupation: the Cypro-Geometric II and III and the Cypro-Archaic I periods (ca. 950–600 BCE), although the absolute dates are still debated [18]. In addition to a Black-on-Red ceramic tradition found throughout the Levant [19], these periods are typified by White Painted and Bichrome Wares, both of which consist of a well-levigated buff fabric with a white slip, on top of which are painted black bands including stripes, geometric circles, and swastikas, as well as stylized palmette motifs; Bichrome Ware is distinguished from White Painted Ware by the addition of red painted bands. Although a wide variety of forms exist on the island of Cyprus, the morphological range in the Amuq is quite restricted. White Painted and Bichrome vessels here consisted primarily of stemmed vertical-sided bowls, barrel jugs, and small juglets [20] (Fig 2). These forms are recognized through parallels with Gjerstad’s typology based on tombs he excavated on Cyprus, since only a handful of complete vessels were excavated in the Amuq.

Because of these strong morphological and stylistic parallels it has always been assumed, though never empirically tested, that White Painted and Bichrome Wares found in the Amuq were imported to that region directly from Cyprus itself. The most plausible arrival point on the mainland would have been the small coastal site of Al Mina in the Orontes River delta, which was founded in the mid-ninth century BCE or slightly later [21,22]. This site produced high quantities of White Painted and Bichrome Ware sherds [23], and in later centuries became a Greek colony dedicated to facilitating trade between the Near East and the Aegean [24,25].

However, the simplicity of this ‘exclusive-import’ model of Cypriot-style pottery on the Eastern Mediterranean littoral is belied by both archaeological findings and analytical studies in nearby Cilicia to the northwest of the Amuq. Excavations at the site of Tarsus-Gözlükule, for example, discovered stylistically clear unbaked Cypro-Geometric pottery inside of a pottery kiln [26]. Likewise, the recent excavations at Kilise Tepe recovered White Painted Ware vessels of the Cypro-Archaic I period, which they date to 750–650 BCE, from the interior of a kiln [27]. These archaeological contexts offer unambiguous evidence for local Cilician production of White Painted and Bichrome Ware vessels that would otherwise have been assigned a Cypriot origin on macroscopic grounds.

Analytical methods of various kinds have since been deployed at other sites in Cilicia to demonstrate the assemblage of White Painted and Bichrome Ware vessels as having been both locally produced and imported from Cyprus. At Sirkeli Höyük, for example, petrographic analysis suggested the use of local clay to produce White Painted and Bichrome Ware pottery recovered at the site [28]. More pertinent to the present study, two projects using NAA on early first millennium pottery excavated from the site of Kinet Höyük have confirmed that at least some of their Cypriot-style ceramics were produced in Cilicia while others were indeed imported, possibly from Cyprus [29,30]. One of these studies [30] had sufficient chronological control to propose that the exchange of ceramics from Cyprus came to a halt with the arrival of the Assyrians in the late eighth century. Ceramics from Tel Dor on the coast of northern Israel have been the subject of another NAA study. Although the sample size was highly limited, the author argues that this site’s Cypro-Geometric sherds show a mix of both imports and local production [31].

The findings at Kilise Tepe and Tarsus-Gözlükule, plus the provenience studies that have been performed at Kinet Höyük and elsewhere, indicate that geochemical analysis of White Painted and Bichrome Ware sherds from the Amuq Valley using pXRF and NAA would be extremely informative for our understanding of ceramic exchange patterns during the early first millennium BCE.

## Portable X-Ray Fluorescence

In order to preserve the Oriental Institute Museum’s collection from the Syrian-Hittite Expedition, initial investigation of the White Painted and Bichrome Wares employed non-destructive analysis with pXRF. Although the application of pXRF to ceramics has been criticized [32], appropriate analytical protocols succeed in generating data that can address specific research questions [33]. In this case, we sought to determine if geochemical analysis supports the assumption that all White Painted and Bichrome Ware pottery found in the Amuq was imported.

### Materials and Methodology for Portable X-Ray Fluorescence

White Painted and Bichrome Ware sherds from Tell Tayinat (n = 77), Çatal Höyük (n = 19), and Tell Judaidah (n = 15) were analyzed with pXRF. Permission for the analysis was provided by the Oriental Institute Museum; there were no permits required because all of the pottery included in this study is the property of the Oriental Institute Museum. The Cypriot-style pottery from Tell Tayinat and Çatal Höyük will soon be published and study of the material from Tell Judaidah is ongoing [16,20]. These samples represent all of the White Painted and Bichrome Ware pottery in storage at the Oriental Institute that could be analyzed with our non-destructive methodology. The collection includes additional sherds that were excluded from this analysis for the following reasons: because of the thinness of the sherd it was not possible to completely cover the receptor of the instrument; encrustation covered the sherd; or the curvature of the sherd made it impossible to take usable readings. In addition, we analyzed contemporary Common Ware sherds from Tell Tayinat (n = 17), selected because the frequency of Common Ware is indicative of local production.

The samples were analyzed with the Bruker Tracer III with Rhodium anode x-ray tube at the Oriental Institute. Analysis was conducted for a 300s count time at 40kV and 11.5μA with a Ti (25μm), Al (300μm), Cu (150μm) filter over an area measuring roughly 7 mm^2^. The section of the sherd, rather than the surface, was targeted to ensure that surface treatments such as slips or painted decorations would not distort the results. Prior to the analysis, the section of the sherd was cleaned using a compressed air canister. Raw data in the form of spectra were generated with S1PXRF software. A Bayesian deconvolution of the pXRF spectra, performed with Artax software, yielded semi-quantitative net peak areas (NPA) for the following elements: As, Ba, Ca, Co, Cr, Cu, Fe, K, Mn, Nb, Ni, Pb, Rb, Sn, Sr, Th, Ti, U, Y, Zn, and Zr. The use of NPA is preferred over quantitative terms such as parts per million or weight percent in this case because it reinforces the semi-quantitative nature of the data while still allowing for the use of multivariate statistics [33,34]. Heterogeneity of ceramic material presents a problem for pXRF as a measurement taken over an inclusion may distort the results. To account for this, three readings were taken for each sherd in different locations and then the NPAs were averaged. As a way of evaluating the internal consistency for the measured elements, coefficients of variance (CV) were calculated for the three measurements taken on each sherd. Ideally CVs would be below 10% for elements used in multivariate analysis; however this would have restricted the analysis to only Ca, Cu, Fe, Rb, Sr, Ti, Zn, and Zr. To expand the suite of elements available for analysis, it was necessary to include all elements with average CVs below 20% (Table 1). This left the following elements for analysis: Ba, Ca, Cu, Fe, K, Mn, Nb, Ni, Rb, Sr, Ti, Y, Zn, and Zr. Both Cu and Ni were excluded from the analysis because they are partially the results of the instrumentation [34]. Hunt and Speakman [35] have called into question the ability of pXRF to measure the Kα peak of Ba because its optimal excitation energy exceeds 50 kV. While our analytical method did not reach this energy level, Ba is a common element in pottery from the Eastern Mediterranean and the NPA for its Kα peak is sufficiently consistent for use in the analysis of this particular dataset.

**Table 1:** Elements measureable with pXRF and CVs calculated for Net Peak Areas.

Elements	AsKα	BaKα	CaKα	CoKα	CrKα	CuKα	FeKα	KKα	MnKα	NbKα	NiKα
CV (%)	29.9	16.4	9.2	20.2	22.5	6.8	6.5	18.5	12.9	16.9	11.7
Elements	PbLα	RbKα	SnKα	SrKα	ThLα	TiKα	ULα	YKα	ZnKα	ZrKα	
CV (%)	21.3	8.2	30.3	5.8	34.2	8.5	77.9	14.6	8.7	8.2	

Multivariate statistical analyses were conducted with the GAUSS 8.0 software developed by MURR. The software converted the NPAs into base-10 logarithms to account for differences in magnitude between major and minor elements and then a combination of Euclidean distance hierarchical clustering, principal component analysis, and Mahalanobis distance tests were used to interpret the geochemical data in accordance with established analytical conventions [36,37].

### Results for Portable X-Ray Fluorescence

Principal component analysis divides the data into two geochemical groups, labeled alpha (n = 75) and beta (n = 32). There remain an additional 21 specimens that could not be assigned to a group. Our statistical approach set aside a priori archaeological information to create groups based solely on geochemical data. It is not uncommon for such methods to leave 20% to 40% of the dataset unclassified [38]. Several reasons may account for ungrouped specimens: the vessel may come from a production locale that was underrepresented in the analysis and, therefore, could not form a statistically robust geochemical group; the analysis may have been contaminated; or the sherd in question may be a statistical anomaly.

**Fig 3:**
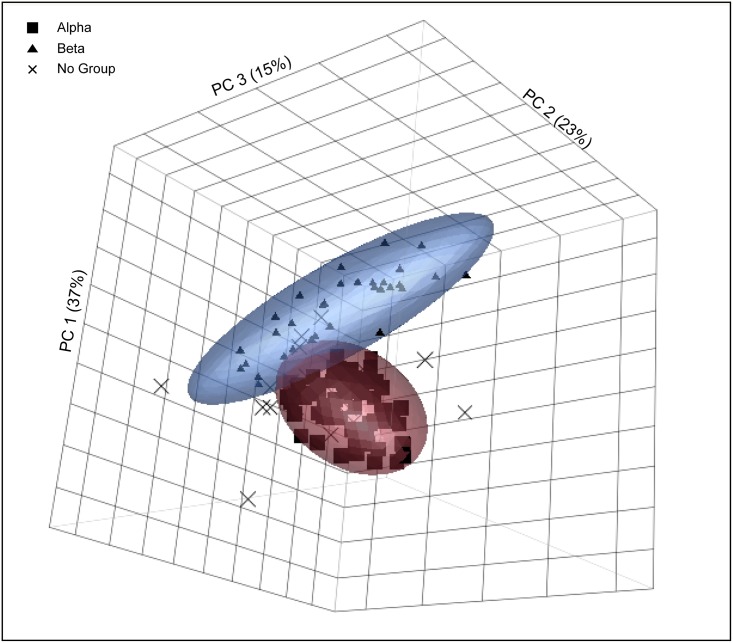
Principal component analysis for the pXRF data. Scatter plot of principal components 1–3. Ellipses are drawn to 90% confidence intervals.

A scatter plot of principal components 1–3, which account for 75% of the cumulative variation, illustrates the separation between alpha and beta (Fig 3). Characteristic of alpha are elevated levels of Rb, K, and Fe while beta has greater concentrations of Nb and Sr (S1 Table). Alpha includes White Painted and Bichrome Ware pottery from the regional capital of Tell Tayinat (n = 51) as well as the secondary settlement of Çatal Höyük (n = 14) and the tertiary site of Tell Judaidah (n = 10). Beta, on the other hand, is found only at Tell Tayinat where it occurs in both the White Painted and Bichrome (n = 16) and Common (n = 16) Wares (Table 2).

**Table 2:** Geochemical groups alpha and beta by site.

	Alpha	Beta	No Group
Tell Tayinat White Painted/Bichrome Ware	51	16	10
Tell Tayinat Common Ware	0	16	1
Çatal Höyük White Painted/Bichrome Ware	14	0	5
Tell Judaidah White Painted/Bichrome Ware	10	0	5
Total	75	32	21

It is possible to draw two conclusions from the pXRF data. First, the White Painted and Bichrome Wares consumed at Tell Tayinat were the result of at least two distinct production locales. The fact that beta includes Common Ware and its distribution is restricted to Tell Tayinat is a strong indication that this geochemical group can be regarded as production centered in the vicinity of the regional capital. The second conclusion that can be drawn is that all three sites had access to alpha, which, given its compositional difference from beta, is unlikely to have been produced in the vicinity of Tell Tayinat and may represent an import from outside the Amuq. It is with the aim of investigating the possible origins of alpha and beta that specimens from both groups were submitted for NAA.

## Neutron Activation Analysis

Whereas pXRF is a relatively new technique, NAA has been the accepted standard for the sourcing of pottery for over half a century. In general, compositional analysis employs two different approaches to sourcing pottery. The first, often referred to as the criterion of abundance, assumes that the majority of pottery found at a site was produced within the vicinity [39]. This approach requires a broad sampling strategy that includes hundreds of samples taken from a variety of wares that often span several occupation phases at a site [30,40]. Given the destructive nature of NAA and the importance of the Syrian-Hittite Expedition collection to future research, it was critical that sampling be minimal, ruling out the possibility of adopting a criterion of abundance approach. The second method for sourcing pottery compares ceramics to geological samples. Unlike obsidian, which comes from a homogenous geological source with a chemical composition that remains unaltered by anthropogenic actions, pottery is a combination of clays and tempers taken from heterogeneous geochemical deposits that have the potential to be modified in any number of ways during production. As a result, a one-to-one match between a geological sample and pottery is highly unlikely and one cannot identify a specific source of clay used by ancient potters. However previous studies have used extensive geological sampling to identify the range of compositional variation found in materials available to potters, and, when this compositional profile compares favorably to the ceramics, a case can be made for locating the production of the pottery within a broadly defined region [41–43]. A rich NAA archive for the Eastern Mediterranean exists, which includes over 150 geological samples from the Amuq, Cyprus, and Cilicia. The comparison of a selection of White Painted and Bichrome Ware sherds to this larger archive allows for the identification of possible sources for alpha and beta.

### Materials and Methodology for Neutron Activation Analysis

Mahalanobis distance tests from the pXRF study identified six samples that can be considered highly representative of each geochemical group (Table 3). Membership probability, as calculated by Mahalanobis distance, is a measure of the likelihood that a sherd randomly selected from the defined geochemical group will have an equal or greater distance than the sherd in question from the geometric mean of the group in multivariate hyperspace [44]. In other words, a sherd with a membership probability of 90% will be closer to the geometric mean than 90 of 100 randomly selected samples that belong to the geochemical group. In two cases (TTY023, TTY010) samples were selected that had membership probabilities below 80%. This was a necessary compromise because the sampling of sherds with higher membership probabilities would have required the destruction of painted decoration.

**Table 3:** Membership probabilities for samples submitted for NAA.

Alpha		Beta	
ANID	membership probability	ANID	membership probability
TTY003	84.7	TTY010	45.1
TTY020	96.6	TTY040	90.9
TTY023	62.7	TTY048	98.5
TTY045	96.9	TTY052	94.3
TTY062	98.4	TTY064	93.3
TTY068	85.9	TTY070	99.1

All membership probabilities are calculated with Mahalanobis distance using principal component scores 1–12 from the pXRF statistical analysis.

We submitted these samples to MURR for NAA. The laboratory generated data with a standard-comparator method that used SRM-1633b Coal Fly Ash, SRM-278 Obsidian Rock, and SRM-688 Basalt Rock. New Ohio Red Clay, an internal reference material, served as a check-standard. The analysis provided concentrations for the following elements: Al, As, Ba, Ca, Ce, Co, Cr, Cs, Dy, Eu, Fe, Hf, K, La, Lu, Mn, Na, Nd, Ni, Rb, Sb, Sc, Sm, Sr, Ta, Tb, Th, Ti, U, V, Yb, Zn, and Zr.

The NAA data for these samples of Cypro-Geometric pottery were compared against archival data from the Amuq, Cilicia, and Cyprus (S2 Table). In 2001 a research team working in the Amuq submitted 349 specimens to MURR for NAA. The focus of the research was ceramic production at Tell Kurdu, a Chalcolithic site located approximately 12 km from Tell Tayinat [45,46]. The study included sediment samples (n = 24), wasters (n = 3), and mud-bricks (n = 2) from 10 sites in the Orontes watershed. Although the Tell Kurdu study remains unpublished, MURR made this data available in accordance with the laboratory’s data maintenance policy. The Anatolian Iron Age Project has generated NAA data for 114 sediment samples taken from across Cyprus [47] as well as an additional 12 sediment samples from the vicinity of Kinet Höyük in eastern Cilicia [30]. The Kilise Tepe team collected an additional 11 sediment samples from the areas surrounding Kilise Tepe and Tarsus-Gözlükule, which they analyzed at the Demokritos Laboratory in Athens [48]. Collectively, these sediment samples provide compositional information for the Amuq and Cyprus as well as the areas surrounding the contemporary sites of Kinet Höyük, Kilise Tepe, and Tarsus-Gözlükule, all three of which were production centers for White Painted and Bichrome Wares.

MURR, the Anatolian Iron Age project, and the Kilise Tepe team generated their data using three different analytical protocols. A previous study by Hein et al. [49] has demonstrated that these differences can result in variations for elemental concentrations; however conversion factors are known that allow for the direct comparison of data collected by these facilities [50]. Each laboratory collects data for different elements and for some elements conversion factors do not exist, restricting the elements available for analysis to the following: As, Ce, Co, Cr, Cs, Eu, Hf, La, Lu, Rb, Sb, Sc, Sm, Ta, Th, U, and Yb. It should be noted that this is roughly half the elements measured by MURR. Such a reduction in the number of analytes has the potential to reduce the resolution of statistically defined geochemical groups. However, this is necessary in order to incorporate the relevant datasets.

Before comparisons commenced, all specimens included in the statistical analysis were normalized with Sc. One concern when comparing pottery to sediments is the dilution effect of quartz, which decreases the measurements for the elements analyzed with NAA [51,52]. In a study conducted on pottery from Portugal, Dias and Prudêncio [53] determined that normalizing data by Sc is a particularly effective means to compensate for the quartz dilution, and Grave et al. [42] found this to be the case as well for the sediment samples that they collected in Cyprus and form part of this analysis. After normalizing the data with Sc, the same statistical protocols were followed as those employed to interpret the pXRF data.

### Results for Neutron Activation Analysis

**Fig 4:**
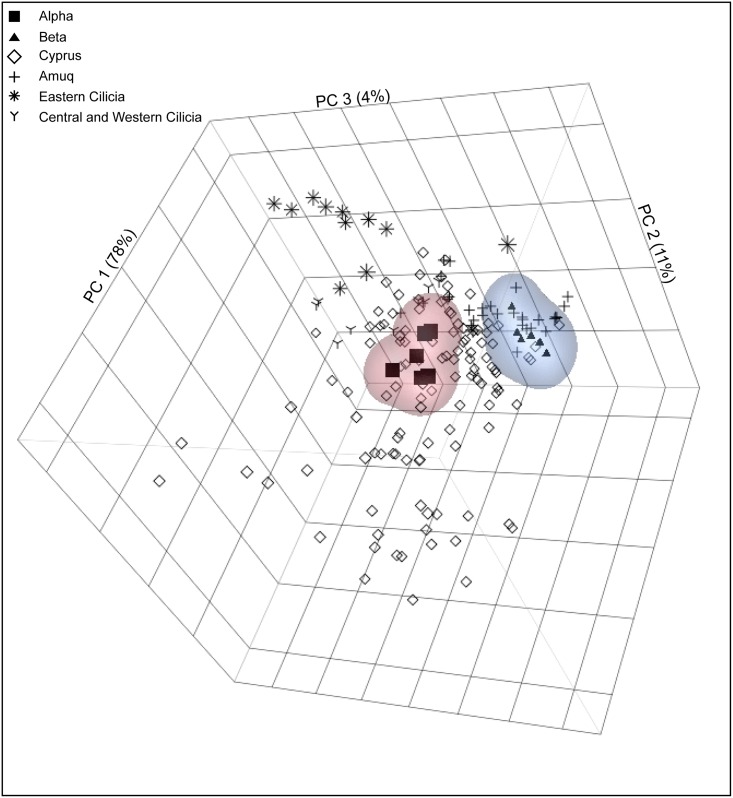
Principal component analysis for the NAA data. Scatter plot of the principal components 1–3. Non-parametric density contours are drawn to 90% confidence intervals.

**Fig 5:**
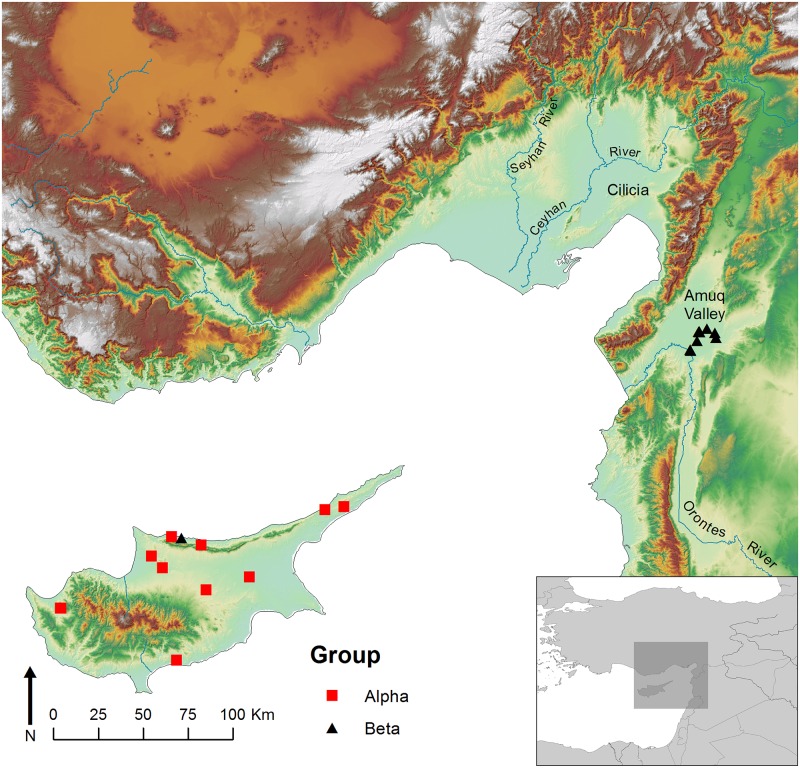
Location of sediment samples that match with geochemical groups alpha and beta.

Principal component analysis reproduces the two distinct geochemical groups initially characterized by pXRF and identifies a number of sediment samples that have compositions similar to that of alpha and beta (Figs 4 and 5). There are 11 sediment samples that plot with alpha, all of which originated in Cyprus. Previous analysis classified all of these sediment samples as being a product of the Circum-Troodos sedimentary succession that occurs throughout central and southern Cyprus [42]. Those samples that group with beta originated almost entirely from the Amuq and several of these samples belong to wasters and sediment samples taken from Tell Atçana, less than a kilometer from Tell Tayinat. The one notable exception for beta is CYP048, which is from the Kyrenia Terrane of Cyprus [42]. The inclusion of this specimen with beta may point to a broadly similar composition between this part of northern Cyprus and the Amuq. It is significant that the samples taken from near the Cilician sites of Kinet Höyük, Tarsus-Gözlükule, and Kilise Tepe do not compare favorably with either alpha or beta. While it remains beyond the ability of geochemical analysis to identify the specific clay deposits used by ancient potters, a comparison of the archival data to the White Painted and Bichrome pottery demonstrates that alpha consists of raw materials originating in Cyprus while potters operating in the Amuq produced beta.

## Discussion and Conclusions

The distribution of White Painted and Bichrome Ware sherds from the Cypro-Geometric and early Cypro-Archaic periods in the Amuq Valley is contrary to what one would expect. Unlike the usual pattern in which pottery types occur with decreasing frequency the further away from the production locale, geochemical group alpha, a close match with sediment samples from Cyprus, far outnumbers geochemical group beta, which aligns with sediment samples taken from the Amuq. In short, our results indicate that inhabitants of the Amuq Valley during the early first millennium BCE imported the majority of their White Painted and Bichrome Ware vessels from Cyprus, a point previously assumed but never tested, and that the locally produced Cypriot-style pottery was supplementary to the imports.

We are not able to explain the distinction between alpha and beta through vessel shape or chronology. With respect to morphology, too many of the samples are body sherds, making a positive identification of the vessel shape impossible in most cases. Regarding the sherds’ dating, the painted patterns are mostly typical of the Cypro-Geometric III period (ca. 850–750 BCE), though several likely date to the Cypro-Archaic I period (ca. 750–600). This is consistent with the stratigraphic positions of the pottery from Tell Tayinat’s palace floor levels that pre- and post-date the Neo-Assyrian Empire’s conquest of 738 BCE [20], although drawing conclusions from stratigraphy is hardly as sound here as with the recent excavations at Kinet Höyük, for example [30]. Unfortunately, the stratigraphic precision of the 1930s excavation is too deficient, and our sample size too limited, to generate a chronological interpretation of the consumption of the alpha and beta groups.

Despite these inevitable shortcomings in the data, there are a number of conclusions that can be drawn about the nature of economic exchange between Cyprus and the Amuq Valley. The first and most obvious of these is that Cypriot imported pottery was widely available throughout the Amuq Valley. White Painted and Bichrome Ware vessels made on the island were identified at all levels of the kingdom of Patina’s settlement hierarchy—capital city, town, and village. This finding indicates a robust economy of interaction between Patina and polities on Cyprus. Although it is not yet possible to identify specifically which Cypriot kingdom (or kingdoms) was the source of the Cypriot vessels, Salamis, Idalion, Kition, or Amathus are all possible contenders for interaction with Patina given the distribution of sediment samples that match with group alpha. This is a topic that warrants further research. Equally significant, none of the sediment samples from Cilicia was a match for either geochemical group, suggesting that the White Painted or Bichrome Ware vessels that are known to have been produced in that region did not make their way to the city-state of Patina. The local polity in Cilicia, ancient Que, seems to have had an independent economic relationship with Cyprus, at least as far as ceramics are concerned.

The second major finding is that a limited proportion of the White Painted and Bichrome Ware vessels were locally produced using clay from the Amuq Valley itself. Similarly, local production of White Painted and Bichrome Ware is attested in Cilicia. However, in the case of the Amuq it is intriguing to note that the locally produced vessels are restricted to the capital city of Tell Tayinat while imports are available at all levels of the settlement hierarchy. This distribution pattern requires explanation, although for now only tentative proposals can be offered.

Previous typological analysis of the pottery recovered from Tell Tayinat has indicated that the vast majority of White Painted and Bichrome Ware vessels at that site are small-medium sized bowls or barrel jugs, that is, vessels for the pouring and consumption of liquids [20]. It is likely, therefore, that these forms were imported from Cyprus to serve as a component of an elite feasting assemblage, one that would have been shared by administrators at other settlements in the city-state such as Çatal Höyük and Tell Judaidah. One possibility for the presence of locally produced vessels at Tell Tayinat alone, therefore, is that the greater number of elites conducting banquets at the capital city drove demand to a point where a supplementary source was necessary. The producer of the locally made vessels is unknown. It is possible that a Cypriot potter or potters established a workshop in the city, although the lack of other indicators of Cypriot presence perhaps argues for production by a local potter from Patina instead.

The findings of this research emphasize the number of questions that remain to be answered regarding economic interaction in the Eastern Mediterranean during the Iron Age. This project has demonstrated that the city-state of Patina in southeastern Anatolia’s Amuq Valley conducted pottery production and trade with Cyprus in a manner distinct even from its immediate neighbors, suggesting that economic behavior among the Syro-Anatolian city-states was highly particular to each kingdom. Further research into the production and consumption of ceramics in this region will hopefully shed additional light on the Syro-Anatolian political economy and the broader Eastern Mediterranean interaction sphere.

## Supporting Information

S1 Table Net peak areas for elements included in the pXRF analysis. (XLSX)

S2 Table Elemental concentrations in parts per million included in the NAA analysis. Conversion factors calculated by Karacic et al. [50] have been applied to this data.
(XLSX)
